# The Future of Pharmaceutical Manufacturing Sciences

**DOI:** 10.1002/jps.24594

**Published:** 2015-08-17

**Authors:** Jukka Rantanen, Johannes Khinast

**Affiliations:** ^1^ Department of PharmacyFaculty of Health and Medical SciencesUniversity of CopenhagenDenmark; ^2^ Institute of Process and Particle EngineeringGraz University of TechnologyGrazAustria; ^3^ Research Center Pharmaceutical EngineeringGrazAustria

**Keywords:** quality by design (QBD), process analytical technology (PAT), mathematical model, materials science, *in silico* modeling

## Abstract

The entire pharmaceutical sector is in an urgent need of both innovative technological solutions and fundamental scientific work, enabling the production of highly engineered drug products. Commercial‐scale manufacturing of complex drug delivery systems (DDSs) using the existing technologies is challenging. This review covers important elements of manufacturing sciences, beginning with risk management strategies and design of experiments (DoE) techniques. Experimental techniques should, where possible, be supported by computational approaches. With that regard, state‐of‐art mechanistic process modeling techniques are described in detail. Implementation of materials science tools paves the way to molecular‐based processing of future DDSs. A snapshot of some of the existing tools is presented. Additionally, general engineering principles are discussed covering process measurement and process control solutions. Last part of the review addresses future manufacturing solutions, covering continuous processing and, specifically, hot‐melt processing and printing‐based technologies. Finally, challenges related to implementing these technologies as a part of future health care systems are discussed. © 2015 The Authors. *Journal of Pharmaceutical Sciences* published by Wiley Periodicals, Inc. and the American Pharmacists Association J Pharm Sci 104:3612–3638, 2015

## INTRODUCTION

Traditionally, the pharmaceutical and biopharmaceutical industries were not the forerunner of innovative engineering solutions and new principles of chemical engineering. For many decades, the manufacturing of drug products were controlled by a regulatory framework that safeguarded the quality of the final product and performed testing of batch‐based operations, raw material and end‐product characteristics, fixed process conditions, and in‐process material. Limitations related to this quality by testing thinking have widely been acknowledged both for small molecule and biopharmaceutical products.^1^, ^2^ In contrast, other fields of processing and related manufacturing sciences have successfully implemented sophisticated technologies to increase our current process and product understanding.

However, over the last years, there has been growing interest in increasing the safety and quality of medications while simultaneously cutting the cost of manufacturing of pharmaceuticals by implementing more structured pharmaceutical development and manufacturing approaches. Especially, the rapidly spreading acceptance of science‐based approaches has created a more flexible environment for implementing already‐existing and well‐established chemical engineering knowledge.^3^, ^4^ A rather recent example is the introduction of the United States Food and Drug Administration (US FDA) process analytical technology (PAT) guidance and the quality by design (QbD) approach by the International Conference on Harmonization (ICH). The QbD‐based thinking is a perfect opportunity for the pharmaceutical community to take the manufacturing sciences into the new millennium. It has to be, however, emphasized that the concept of PAT is not entirely new, as process analysis/control has been an important area of chemical engineering for decades.^5^, ^6^ Nevertheless, PAT introduced the idea of real‐time process control and real‐time quality assurance (QA) in pharmaceutical manufacturing, being the basis for modern process engineering. An example of it are novel manufacturing methods (e.g., based on continuous flow chemistry) that are now being introduced by industry, academia, and regulators.^7^, ^8^, ^9^ The recently published white paper series from the MIT‐Strathclyde symposium on continuous manufacturing (CM) in 2014 highlights the current state of thinking.^10^, ^11^, ^12^, ^13^, ^14^, ^15^, ^16^, ^17^, ^18^ Moreover, the ICH is in the process of developing a new guideline (ICH Q12) that can serve as basis for implementing CM across the industry in a widespread manner.

The use of QbD terminology, including such abbreviations as QTPP (quality target product profile), CQAs (critical quality attributes), and CPP (critical process parameters), is deliberately minimized in this review. Although it is important to understand these concepts, especially QTPP from a patient point of view, when implementing QbD into practical use, this review rather intends to cover the underlying science, introduce the main techniques involved in the QbD approach, and provide an overview of future challenges. One related yet extremely difficult to define concept is process understanding. When do we completely, or even partially, understand a process or a single unit operation completely? Does it happen after implementation of a simple experimental design containing four experiments or only after a full risk analysis coupled with first principles physical modeling? Or are we aiming at *ab initio* molecular modeling approaches to enlighten molecular level phenomena during operations? As the level of process understanding is case specific, this review is organized around the practical tools and has the objective of providing an overview of these tools together with future perspective.

One visible part of all PAT and QbD activities during the past decades has been sensor development.^19^ In many cases, near infrared (NIR) spectroscopy has been used almost as a synonym for PAT. Note that science‐based manufacturing of pharmaceuticals involve not only application of novel process analytical sensors and measurement solutions, but also the utilization of other fundamental tools for increasing our understanding by implementation of risk management strategy, formalized design of experiments (DoE), advanced data analysis techniques, first‐principles based process modeling and control, and fundamental material characterization together with molecular modeling.

These fundamental tools of science‐based manufacturing are not part of a standard pharmaceutical teaching curriculum and, in the future, special attention should be paid to identifying the elements that should be introduced into pharmaceutical education. As consequence, the future development of the elements of pharmaceutical engineering in various educational programs requires special attention. This “step forward” in education is also needed to safeguard the development of a regulatory framework, as several emerging areas of manufacturing are still not generally accepted or even fully defined. The concept of CM provides us with a fascinating opportunity to revise the entire idea of a traditional batch operation. Although continuous operations are well defined and exist in the field of chemical engineering sciences, their implementation in the pharmaceutical context requires fundamental research. Another important concept is the implementation of real‐time release, which requires a sound combination between manufacturing sciences and a new type of thinking in the fields of analytical sciences and risk management. Moreover, current developments in process validation emphasize the need for implementing the QbD thinking.

Prescribing medicine today is based on a “one size fits all” principle. However, more personalized (combination) solutions in several critical therapy areas are required. The latest developments in genomics and diagnostics have enabled the advent of new innovative drug products relying on a combination of diagnostic tools and personalized dose. All this paves the way to a future health care system based on personalized medicines, as recently outlined in the precision medicine initiative (PMI).^20^ The current level of innovation in dosage form design and manufacturing of these products cannot meet the needs of personalized medicine. As such, novel manufacturing solutions, enabling the flexible manufacturing of personalized dosages, are required.

**Figure 1:**
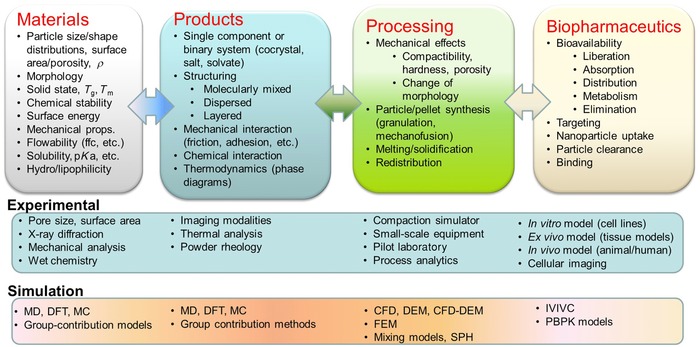
Engineering view of pharmaceutical development (MD, molecular dynamics; DFT, density functional theory computations; MC, Monte Carlo methods; CFD, computational fluid dynamics; DEM, discrete element method; FEM, finite element method; SPH, smoothed particle hydrodynamics; IVIVC, *in vitro–in vivo* correlations; PBPK, physiologically based pharmacokinetics).

In summary, we are currently observing a change in the paradigm change, with engineering principles and product design becoming the guiding principle of pharmaceutical development. That is, we are adopting a way of thinking, according to which pharmaceutical ingredients, pharmaceutical products, the related manufacturing processes, and the biopharmaceutical properties are considered simultaneously and quantitatively. Figure 1 demonstrates this engineering view of pharmaceutical development.

We have to understand the compounds and materials, predict and/or measure compound properties, and define and characterize their constitutive behavior. Moreover, we have to understand how ingredients interact (thermodynamics vs. kinetics) and how the delivery requirements determine the ingredients and the corresponding processing. With regard to the process, we must understand and identify the critical variables and their effect on quality and develop and validate mathematical models, which largely contributed to the successful operation of chemical and petro‐chemical plants. Most importantly, however, the patient has to be the center of focus.^21^

This review aims to cover the recent developments in the manufacturing sciences related to QbD‐based thinking and to outline the future direction of scientific research in this field, supporting a further development of the regulatory framework.

## FUNDAMENTAL TOOLS FOR INCREASED PROCESS UNDERSTANDING

### Risk Management and DoE

#### Risk Management

Quality risk management (QRM) can be defined as an integrated action aiming at, first, identifying, assessing and prioritizing risks and, second, at minimizing, monitoring, and controlling the related undesired event. Evidently, QRM is most effective when applied throughout the entire life cycle of a pharmaceutical or bio‐pharmaceutical product. RM is widely utilized in various industries, and several approaches exist. In the QbD context, QRM related to the development and manufacturing of pharmaceuticals with a special focus on customer (i.e., patient) health and safety is important. In practice, all risk management activities should be performed by a team that has enough background to analyze the given product and related processing. This multidisciplinary team should have participants with experience in dosage form design, manufacturing, process engineering and quality functions, and a moderator who can formally lead the risk management process. Risk management is a continuous process and, in many cases, an iterative operation. Based on the existing supporting standards^22^, ^23^ and guidelines,^24^, ^25^ the proper use of risk assessment tools and methods is a daily routine.

**Figure 2:**
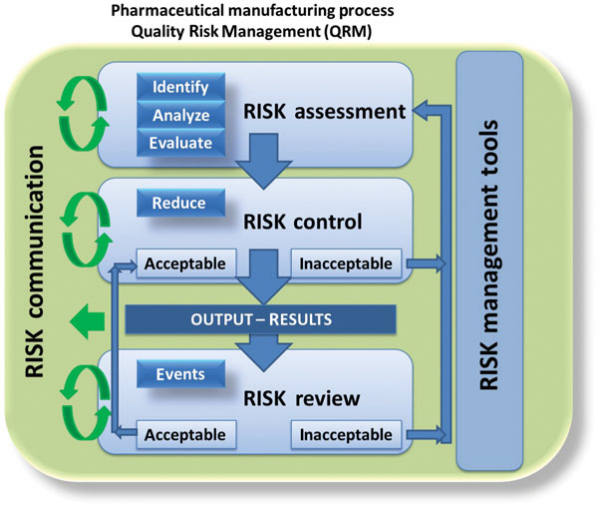
Overview of a pharmaceutical quality risk management (QRM) system.

Risk is defined as a combination of probability of occurrence and the severity of harm.^26^ The QRM workflow consists of (1) initiation, (2) assessment, (3) control, (4) review, and (5) communication of risks, as shown in Figure 2. The assessment involves the identification of hazards based on a systematic use of information. Then, an analysis links the likelihood of occurrence and detectability with the severity of harm during a qualitative or quantitative process. Finally, risks are evaluated and ranked according to defined criteria. Eventually, the risk must be reduced to an acceptable level (control). Here, recommended actions are defined to decrease the severity, probability, and detectability of harm. The goal is to reduce the quality risk to a non‐critical level or to implement decision loops that ensure keeping the risk under control. The QRM workflow considers mechanisms that monitor its output in the review phase. The frequency depends on the level of risk.^27^ Finally, risk must be communicated to various stakeholders (i.e., executive company representatives, authorities, doctors, and patients).

A good starting point is an Ishikawa (fish bone) diagram, which provides an overview of the system under investigation and often minimizes the possible misunderstandings in a multidisciplinary risk management team. The next level of risk management is a more detailed risk assessment instrument. There is a variety of generally accepted tools and the selection should be based on the formal in‐house risk management expertise. It is important to remember that the depth of a risk assessment depends on the state of development, that is, approaches to the first‐in‐man formulation are different from those applied to commercial production. In the pharmaceutical manufacturing environment, mainly tabular risk analysis methods are used to support plant or equipment qualification,^28^ process,^29^, ^30^ method,^31^ cleaning^32^, ^33^ or computerized system^34^, ^35^ validation, service, and maintenance.^36^ These QRM tools also support the good manufacturing practice (GMP) or good engineering practice. The most commonly used methods and tools in risk management recommended by the ICH in the Q9 “QRM” guideline are:

- Risk ranking and filtering
- Preliminary hazard analysis‐criticality assessment
- Fault tree analysis
- Failure mode and effects analysis (FMEA)^37^, ^38^
- Hazard analysis and critical control points^39^
- Hazard and operability analysis^40^

One of the most widely accepted risk analysis tool is FMEA, which enables quantitative evaluation of possible risk scenarios. Recent published examples of its use in pharmaceutical manufacturing include optimization of coating,^41^ mixing,^42^ and spray drying^43^ operations. It should be mentioned that relatively demanding quantitative methods, such as FMEA, are not an ideal starting point for the first risk assessment efforts or for evaluating early development phases.

A successful implementation of risk management comprises not only the risk‐based specification of qualification measures, but also the definition of means to control the risks relating to product quality and process performance. This includes the prevention of failure modes caused by computerized systems. Furthermore, the control and monitoring of CPPs depends on the assessment's outcome. Risk assessment leads to the definition of preventative maintenance and repair activities, such as scheduling of the calibration interval for equipment, which directly affects product quality. The output must be integrated into standard operating procedures.

Quality risk management is essential for the effectiveness of a pharmaceutical quality system as it ensures transparency throughout the product's life cycle. However, today QRM in the manufacturing environment is limited, not only by a selective (and mostly qualitative) use of risk analysis tools in the fields of qualification, validation, service, and maintenance, but also by current risk communication approaches. Moreover, inaccessibility of knowledge, which is stored in paper documents, locally stored files and in employee's heads, is a limiting factor in modern QRM. In addition, QRM is applied only to specific aspects of development or manufacturing. Integrated life‐cycle QRM is largely absent.

In the future, the pharmaceutical industry has to enforce a more integrated and holistic quality‐ and design‐oriented product and process development environment. Practicable strategies and solutions for an efficient knowledge transfer and data handling have to be employed. It is the responsibility of academia, industry, and regulators to provide them, which requires concepts for resolving the lack of data management and providing opportunities of prospective and retrospective consideration. One possible approach with that regard are ontologies^44^, ^45^ or other knowledge management tools. Another future challenge in manufacturing science is improving communication within the QRM framework. Lastly, mechanistic models and simulation tools as quantitative and objective approaches in QRM are largely under‐used. However, efforts^46^ to apply simulation as QRM tool for risk ranking have recently been reported.

#### Design of Experiments

Investigation of the variables that affect processing can be performed using a formal experimental design. Risk analysis should always be the starting point for allocation of the resources for this activity. Without knowledge‐based exclusion of variables, the number of experiments can increase dramatically. It is also important to use prior knowledge to define the range within which the experiments are performed and to exclude experimental areas in which it would be impossible to operate. Utilization of prior knowledge is crucial for ensuring that only a reasonable number of experiments are performed.

A simple set of screening experiments provides a good experimental overview of the system under investigation. A decision on the number of variables to be included and the number of levels at which they are to be investigated will determine the final number of experiments performed. In a simplified case when two variables are investigated at two levels, a relatively low number of experiments are required (four). A number of experiments when applying a full factorial design at two levels can be generalized into a simple equation 2*^k^*, where *k* is the number of variables. However, four experiments are rarely enough even for screening purposes, and experimental activities can easily expand (three/four/five variables on two levels will result in 2^3^/2^4^/2^5^ = 8/16/32 experiments, respectively). Full factorial design enables the investigation of both main and interaction effects but, as mentioned above, with an exponential increase in the cost of experimental activities.^47^ The number of experiments can be reduced systematically by implementing fractional factorial design, with the experimental load calculated as 2*^k^* ^‐*p*^, where 1/*p* is the size of fraction. For example, Andersson et al.^48^ aimed to optimize early drug development tablet formulation by creating a model with a high predictive power and performing as few experiments as possible. The authors highlighted the importance of considering the number of experimental points when the availability of a drug substance is a limitation and utilized a fractional factorial design to minimize the number of experimental runs in their study.

**Figure 3:**
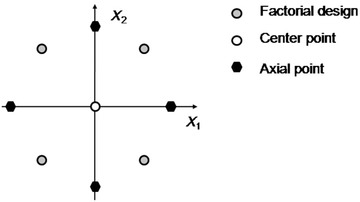
Constructing central composite design (CCD) for two variables.

Design of experiments can further be used for optimization and robustness testing of the operational variables. Factorial design on two experimental levels does not allow modeling of quadratic terms (i.e., possible non‐linear relationships), which can be solved by systematically adding experimental points to the design. By adding a center point (or points, in case of repeated experiments) to the center point and axial points, this problem can be solved and more complex interactions can be modeled by implementing central composite design (CCD; Fig. 3). As with factorial designs, the number of investigated factors can be increased but at the cost of increased experimental load. This can be solved by using fractional factorial design as a starting point for CCD.

Several other experimental designs are available, but the basic idea of adding experimental points in a rational way is still the same and the only difference between these approaches is the number and relative location of these experimental points. For example, in a Doehlert design, in the simplest case experimental points form a hexagon. Experimental points should be selected so that they properly cover the relevant experimental space ensuring the construction of a proper design space on a statistically robust basis. Replicating a given experimental design and repeated experimental points can be used to explore the effect of difficult‐to‐control‐factors, such as a change of the operator, a variation in weather and wear/change of equipment.

The application of different DoE techniques as a part of a science‐based manufacturing approach is widely represented in the literature.^49^, ^50^ Currently, there are several commercial software packages available for both choosing a suitable design and supporting the statistical analysis of the results. Response surface methodology is a classical tool for visualizing the influence of selected variables on a selected response(s). Visualization of the experimental results can be performed, for example, by using contour plots and providing a fast overview of a particular case. This feature is often a built‐in functionality in commercial software packages. There is a variety of software solutions for DoE, from products with Microsoft® copy‐paste logic to statistical programs requiring expert level programming skills. Investing in a solution that requires a more skilled user allows modification of the developed models. This more detailed analysis of the achieved results typically pays back later. Often, a practically feasible solution is to use relatively easy program at the scientist level and more dedicated software solutions for the company's statistics expert.

Classical models explaining the relationship between variables and the observed quality characteristics are based on the ANOVA. The increasing amount of information resulting from a typical DoE may require more efficient algorithms for the development of a model. By implementing multivariate statistics via, for example, GEMANOVA approach,^51^ a more detailed and simple visualization of experimental data from pharmaceutical systems can be achieved.^52^ Non‐experimental approaches based on analysis of historical batch data have also been suggested as a data‐driven support tool for identifying critical process variables.^53^ The orthogonal projection to latent structures approach has been applied to investigate the complex relationships between material characteristics and final product performance.^54^

The complex nature of pharmaceutical materials often requires the utilization of non‐linear modeling in the analysis of experimental results. Other types of modeling based on artificial neural networks, fuzzy logic, and neuro‐fuzzy modeling have been suggested to solve this problem. Analyzing pharmaceutical materials and drawing conclusions based on analytical results is often experience based and cannot always be documented precisely. The pioneering work of Hussain et al.,^55^ Yliruusi and colleagues,^56^ and Leuenberger and colleagues^57^ in the field of artificial intelligence (AI) indicated that this type of modeling can be used for interpreting the results of the experimental design. There are several examples of improved interpretation of experimental data based on AI.^58^, ^59^ Fuzzy logic can also be implemented for mimicking the process of human decision‐making and handling visual information numerically.^60^ AI‐based models can be a part of an overall knowledge management solution and are extremely useful for data mining, that is, for extracting knowledge in the form of linguistic rules from large experimental data sets.^61^, ^62^ One of the key challenges with that regard is the overall knowledge management structure.^63^

### Mechanistic Process Modeling

In the last years, the mechanistic modeling of pharmaceutical unit operations has made significant progress. Many groups, both in industry and academia, have recognized the potential of modern process modeling, including the ability

- to improve the fundamental scientific understanding of a process. In this case, models do not necessarily have to provide an accurate description of the process. Often, qualitative information of the effect of parameters on the system behavior (i.e., via a “learning model”) can suffice.
- to optimize, scale‐up or transfer a process from one equipment to another. In this case, models have to accurately represent the reality.
- to provide quantitative measures in the context of QRM (e.g., FMEA) by performing sensitivity studies (e.g., which parameter is a CPP).
- to study the effect of uncertainty and variability of the material parameters on the process performance.
- to replace experiments during a process characterization phase.
- to study the effect of process disturbance or start‐up and shut‐down phases on the process performance. In this case, transient models are required to capture the process dynamics. Such models can also be used in control systems, for example, for model‐predictive control.

Because of a larger number of simulation tools currently in use, only a limited overview is provided here that only focuses on modeling and simulation of

- fluidic systems including multiphase flows (e.g., bioreactors, synthesis processes, crystallizers, etc.)
- particle‐based processes (e.g., particle handling, powder mixing, etc.)
- fluid‐particle systems (e.g., fluidized beds, suspensions, and particle transport)
- pharmaceutical flow‐sheet or process modeling (e.g., for continuous processes, control models, and global optimization)

#### Fluidic Systems and Multiphase Flows

Computational fluid dynamics (CFD) are well‐established tools for the simulation of pharmaceutical unit operations that involve fluidic and multiphase systems, including stirred tanks, crystallizers, gassed batch reactors, bubble columns, and bioreactors. Generally, the goal is to understand in detail the mixing dynamics, the effect of mixing on the selectivity of competing reactions, the influence of gassing and stirring on the oxygen distribution, the identification of dead zones, or the characterization of the shear rate distribution for shear‐sensitive products. Typical CFD methods include Reynolds‐Averaged Navier–Stokes (RANS) solvers with turbulence modeling of various level of detail.^64^, ^65^ For example, the impact of agitation and shear stress in various types of laboratory equipment (rotator, orbital shaker, magnetic stirrer, and vortex mixer) on the stability of proteins was investigated via RANS CFD methods by Bai et al.^66^ A recent review of CFD in biotechnology was published by Sharma et al.^67^ In the case of fast reactions (or precipitation), mixing models have to be incorporated to model the effects on a scale smaller than the grid size. To that end, probability density function‐based micro‐mixing models can be utilized, which approximate the fluctuations of the species concentrations on the sub‐grid‐scale^68^ (e.g., a RANS CFD model combined with population balances and a micro‐mixing model of impinging jet crystallizers proposed by Woo et al.^69^).

More advanced approaches include large eddy simulations (LES) and direct numerical simulations (DNS). The former method only resolves the evolution of the large‐scale motions by applying a filtering process to the conservation equations of the liquid phase. The resolved flow can be interpreted as a low‐pass filtered representation of the real flow. The effect of the residual motion that resides on scales smaller than the filter width is modeled using sub‐grid‐scale models, for example, the Smagorinsky model.^70^ It was demonstrated that the scheme can accurately predict turbulent hydrodynamics in single‐phase system^71^ and multiphase systems.^72^, ^73^ For example, Marchisio^74^ used LES to simulate particle formation in a confined impinging jets reactors using LES coupled with a sub‐grid‐scale mixing mode. In contrast, DNS does not require any modeling as all turbulent structures are resolved down to the smallest scale. However, with regard to typical engineering applications, due the vast number of grid points required DNS is still infeasible using current computational technology. However, new technologies, such as quantum computing, may solve the complex flow problems under DNS.

**Figure 4:**
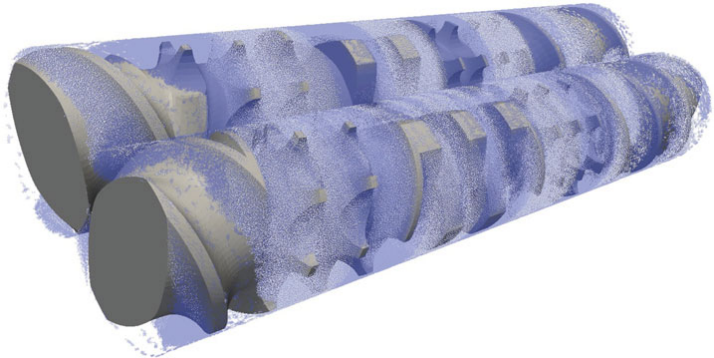
Snapshot of a 3D free‐surface flow in a co‐rotating twin‐screw extruder via smoothed particle hydrodynamics (SPH). The image shows two fully filled intermeshing screws with conveying and mixing elements. The polymer melt is shown as blue and white particles with identical properties, representing the flow. Energy dissipation, flow rate, power consumption, mixing performance, and pressure characteristics can be determined from the simulations.

Another set of methods to solve the Navier–Stokes equation are particle‐based [e.g., the Lattice‐Boltzmann method (LBM)]. The LB scheme employs a simple form of the Boltzmann kinetic equation to recover the macroscopic hydrodynamic behavior of fluids.^75^ The main idea is that fluid flow, which is governed by conservation laws, can be simulated by a many‐particles system obeying the same laws. A set of (fictitious) particles residing on a lattice moves to neighboring sites and exchanges momentum (i.e., by colliding) with particles coming from other directions. The collision rules and the topology of the lattice are defined in such a way that the Navier–Stokes equations are recovered.^76^ Another recently emerging method is smoothed particle hydrodynamics (SPH), a Lagrangian, particle‐based approach that approximates the continuum equations of fluid mechanics based on an interpolation technique for spatially disordered nodes. With this grid‐free method complex free‐surface flows can be captured. A recent example is the simulation of the complex flow in a co‐rotating twin‐screw hot‐melt extruder for pharmaceutical applications^77^, ^78^ (a snapshot is shown in Fig. 4).

**Figure 5:**
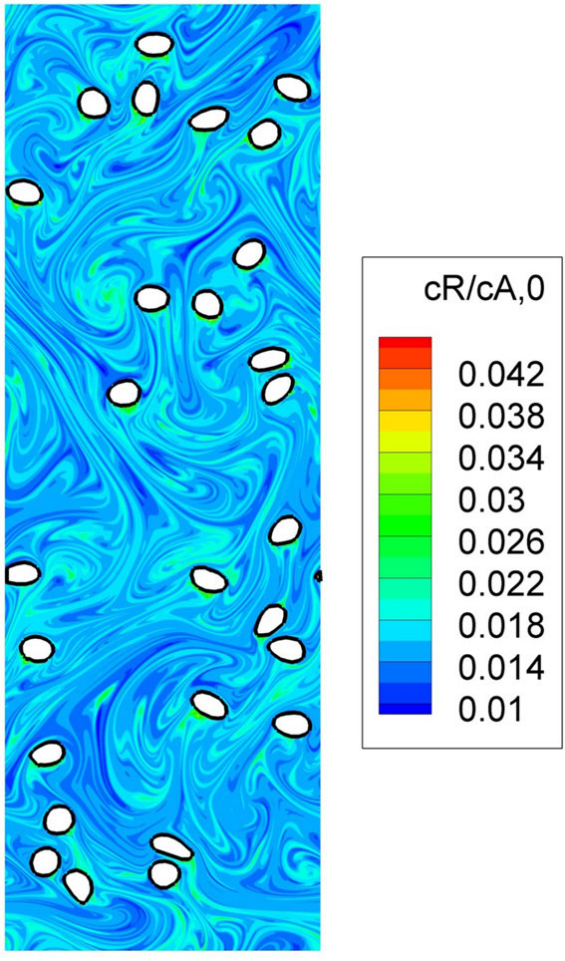
Direct numerical simulation (DNS) of a swarm of fully deformable reacting bubbles, including mass transfer. Reynolds number = 38, Schmidt number = 50. The figure shows the concentration field of gas transferred from bubbles into the gas phase. The complex concentration distribution (striations) in the liquid phase can be seen.

Similarly to single‐phase simulations, a variety of methods can be used to describe flows in multiphase reactors, which are typically based on RANS or LES descriptions of the continuous phase. Currently, the most detailed methods allow the analysis of the deformation of individual bubbles, which in this text are referred to as multiphase DNS (MDNS) as they typically involve DNS of all the phases.^79^ These techniques include the volume of fluid,^80^ Lagrangian methods (where the grid follows the gas‐liquid interface, e.g.,^81^ and^82^) and the front‐tracking method introduced by Unverdi and Tryggvason.^83^ The latter method has been applied by numerous groups^84^, ^85^, ^86^ to study mixing effects including chemical reactions, for example, reactive bubble swarms (∼100 bubbles) with fully resolved deformable and dynamic interfaces^84^ (Fig. 5).

Another approach is the Euler–Euler (EE) method, which treats the involved phases as interpenetrating continua. Its advantage is that, as there are no particles or bubbles, the number of particles is not a limiting factor. However, the interface between the phases is not resolved and, consequently, sophisticated closure models that predict the local bubble size and momentum/mass exchange are required to correctly describe the interaction between the involved phases. Often, a population balance equation (PBE) has to be solved in conjunction with mass, momentum and, possibly, the energy balance, which is computationally demanding. In addition, it can cause instability in the solution procedure and is still a topic of active research as breakage and coalescence kernels cannot be predicted from theory alone and remain somewhat of a fitting parameter.^87^, ^88^

The last major method is the Lagrangian particle tracking, which tracks the dispersed phase, for example, the individual bubbles or droplets, in the flow field as point sources. As the motion of the continuous phase is solved on an Eulerian frame of reference, it is often referred to as Euler–Lagrange (EL) approach.^89^ It was first applied to gas–solid flows in the mid‐90s.^90^, ^91^ With regard to the EL approach, DNS of the continuous phase, that is, a full resolution of all length scales^92^ and the filtered Navier–Stokes equations^93^ have been reported.

#### Particle‐Based Systems

Many pharmaceutical manufacturing operations, especially in secondary (drug product) manufacturing, deal with particles. Examples include powder blending, granulation, milling, roller compaction, tableting, and tablet coating. Depending on the properties of the material, the granular flows can be highly complex, containing arbitrarily shaped particles of various sizes, mechanical attributes, and concentration. Although for many years only continuum approaches prevailed (based on soil mechanics for quasi‐static flows or on kinetic theory for granular flows in the collisional regime), recently new modeling techniques became available to a wider community, which allow a mechanistic simulation of particulate flows. In these methods, particles are considered individual elements and collision forces and the resulting particle trajectories are determined for each collision or time step. There are two main methods: the hard‐sphere approach (assuming binary, instantaneous collisions) and the soft‐sphere approach (allowing multiple and enduring contacts that are modeled by assuming an overlap of particles) that is commonly referred to as the discrete element method (DEM). Although the former is suitable for dilute flows (such as powder conveying) with few collisions, the latter is applied to dense powder flows,^94^ which are typically encountered in pharmaceutical manufacturing. Note that these methods do not consider the gas phase and filling inter‐particle voids.

In DEM simulations, linear (Netwon's second law) and angular momentum balances in all three coordinate directions are solved for every particle. The most critical aspects are the contact detection and the particle interaction model that is used to calculate the forces acting on individual particles during their collisions with other particles and/or walls. Each of these two contact types can be resolved using the same contact model, and the material properties (Young modulus, Poisson ratio, coefficient of restitution, and friction coefficients) for each contact type may differ, such that various materials can be modeled. The forces are used to calculate (linear and rotational) accelerations, which are then integrated in time to compute each particle's velocity, rotation speed, and location. One of the simplest and most commonly used normal and tangential force models are the linear and dashpot and a Hertzian (non‐linear) spring model. However, models allowing the description of plastic deformation also exist. For a more detailed review see Ref. 94.

**Figure 6:**
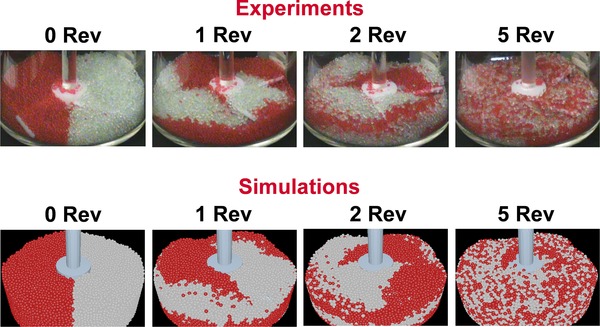
Comparison of experiments and DEM simulations of a powder blending process of cohesionless particles.^95^ In both experiments and simulations, a four‐bladed powder mixer was filled with identical 2 mm Dragonite® glass spheres. One half of the bed was colored red and the particle mixing process was followed for several rotations of the stirred. A good agreement between experiments and DEM simulations was observed.

In recent years, the DEM has widely been applied to improve the understanding of particulate processes in the pharmaceutical industry.^94^, ^95^, ^96^ For example, blending processes were studied by Remy et al.^97^ to understand the effect of blade orientation on particle flow patterns and mixing kinetics. In another paper,^98^ they used experiments and simulations to quantify the effect of varying particle roughness on the granular flow of cohesionless particles and the effects of varying blade speed (see Fig. 6). Zhou et al.^99^ investigated the effects of the blade speed, the particle size, the volume fraction, and the particle density on the segregation of binary mixtures. In addition, Zhou et al.^100^ performed a microdynamic analysis of the particle flow and, especially, the effects of sliding and rolling friction coefficients on three‐dimensional (3D) recirculating particle zones. Continuous blending in a convective mixer was studied by Sarkar and Wassgren.^101^, ^102^ Radl et al.^103^ examined the mixing characteristics of wet granular matter and observed better mixing rates and performance compared to dry granular matter under the same conditions. Radeke et al.^104^ recently presented a GPU‐simulation based approach and studied blending of up to 8 million particles. DEM simulations of a tote blender for performance improvement were recently performed by Ren et al.^105^ DEM simulations of powder blenders were reported to be the basis for design space definition of a blending process within the QbD framework.^42^

**Figure 7:**
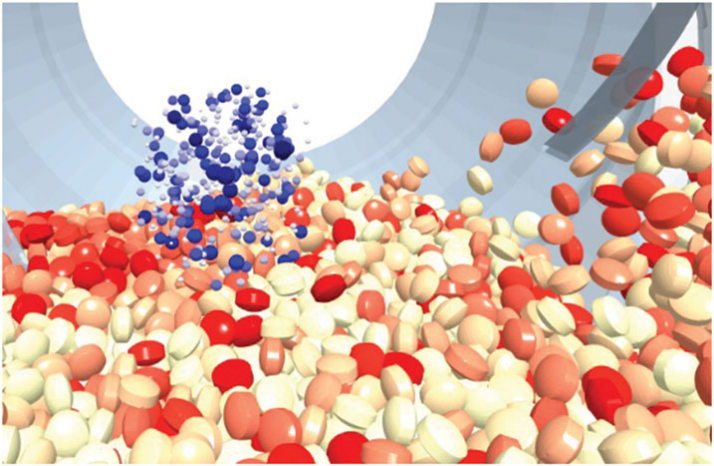
DEM simulation of a tablet coating process, including the coating spray. The spray droplets are colored blue, with darker shades indicating bigger droplets. The tablets are colored according to coating mass, from white (no coating mass) to red (high coating mass). For more details of the simulation setup, refer to Toschkoff and Khinast.^114^

Drum coating processes were extensively studied using DEM (see Fig. 7). With that regard, DEM can provide information about the movement of individual tablets and the duration and frequency of tablet appearance in the spray zone. The number of tablets involved in the process is high enough to have granular behavior, yet in many cases, it is small enough to be handled well using the available computational hardware. For example, Pandey et al.^106^ investigated the movement of spherical particles in a pan coater via DEM. Sahni et al.^107^ used both simulations and experiments to consider the influence of various parameters on mixing in a pan coater. In many simulation studies, to reduce the computational effort the particles either have spherical shape or are approximated by spheres. Few studies focus on the influence of the shape itself.^108^, ^109^ In these cases, the glued‐sphere method is employed to approximate arbitrary shapes by a number of spheres.^110^ An alternative new approach is to apply a contact detection algorithm that models the bi‐convex tablet shape via parts of intersecting spheres.^111^, ^112^ The shape is especially important for intra‐tablet uniformity, as non‐spherical tablets may not achieve perfect uniformity even if the coating time were infinite.^113^

To study such attributes as (inter and intra) coating uniformities, one must know which tablets are in the spray region (i.e., receiving coating mass) at a given time and which are not. Various approaches have been proposed to determine that. Freireich et al.^115^, ^116^ calculated intra‐particle coating variability using a DEM simulation of a rotating cylinder coater. An experimental and computational study of the inter‐tablet coating variability was performed by Kalbag and Wassgren.^117^ The bi‐convex tablets from experiments were approximated in the DEM simulations by spheres of the same volume, since they were reported to have nearly identical circulation times.^118^ Using the output data of a DEM simulation, spray zone detection can be carried out based on the fill fraction of static cubical voxels.^119^ Toschkoff et al.^109^ studied the effect of three fill volumes on the residence time of the tablets under the coating spray, leading to a quantification of the inter‐tablet coating variability for each particle shape. Moreover, Toschkoff and colleagues^114^, ^120^ investigated the impact of different spray models on the simulation results (see Fig. 7). Dubey et al.^121^ investigated the effects of pan speed, fill level, and design of the spray pattern on the coating variability of tablets coated in a rotating pan.

Moreover, other processes were investigated via DEM simulations, including ball milling,^122^ powder rheometry,^123^ hopper flows,^124^ or powder sampling.^125^ An integrated approach to simulating pharmaceutical powder processed was recently presented by Rogers et al.^126^

#### Fluid–Solid‐Particle Systems

Solid particles that are fluidized or suspended in a liquid are frequently used in pharmaceutical manufacturing, for example, in fluid bed drying, agglomeration and coating processes, wet milling, dissolution, suspension production, as well as in the transport of solids. Liquid‐particle systems (e.g., suspensions) and gas‐particle systems behave quite differently and require different simulation approaches. With that regard, we focus on dense fluid (liquid and gas) particle suspensions (i.e., particle volume fractions ϕ_p_ up to the close‐packing limit). A recent review of simulation methodologies for dilute suspensions (where particle–particle collisions are relatively rare but turbulence is important, which is generally the case for ϕ_p_ ≪ 0.01), can be found in Toschi and Bodenschatz.^127^ Most of the methods mentioned below have been applied only to mono‐disperse spherical particles. Studies on irregular particles are scarce and limited to simple shear flow simulations of particles without interstitial fluid.^128^, ^129^

##### Direct Numerical Simulation

Direct numerical simulation provides the most general description of the two‐phase system, with the lowest level of additional models needed. If the true shape of the particles is well approximated, direct simulations can be treated as the ultimate description of the suspension flows and do not necessarily require validation via experimental data. Furthermore, since any additional model for non‐hydrodynamic interaction between particles (e.g., van der Waals or electrostatic forces and the effect of Brownian motion) can easily be incorporated, direct simulation became one of the most powerful tools in the field of suspension mechanics, effectively replacing experiments in some areas (e.g., for microstructure analysis, rheological characterization). Because of the extremely high‐resolution requirements, direct simulations are currently limited to *O*(10^3^) particles. Studies of larger systems are rare^130^ and require significant computational resources. Below we describe three widely accepted methodologies, which have been used primarily to study the rheology of liquid‐particle suspensions (i.e., systems with a density ratio close to unity). They are also applicable to gas‐particle flows and dense bubbly flows.^131^ A more detailed review of suspension mechanics was published by Stickel and Powell.^132^

- *Stokesian dynamics (SD)*: This approach provides the most accurate description of the suspension flow at zero Reynolds numbers, using an analytical solution to the flow of the interstitial fluid.^133^ This is possible because at zero Reynolds number, the equation of fluid motion becomes linear. Hence, SD is intrinsically limited to systems with negligible fluid inertia, that is, the particle Reynolds number must be much smaller than unity. Sierou and Brady^134^ significantly improved SD by increasing the computational efficiency of solving the Stokes flow problem.
- *LBM*: Incorporating the Lattice‐gas‐based algorithms for studying the fluid flow, LBM introduced by the group of Ladd^135^, ^136^, ^137^, ^138^ was widely applied to study suspension flow. LBM‐based simulations can be used for arbitrary Reynolds numbers and provide a significant advantage over SD. LBM was recently applied to study suspension flow by Derksen and colleagues,^139^, ^140^, ^141^ van der Hoef and colleagues,^142^, ^143^ and Hölzer and Sommerfeld.^144^, ^145^
- *Immersed Boundary Method (IBM)*: IBM refers to an approach that resolves the flow around individual particles via a “classical” Navier–Stokes solver (typically on a regular computational grid) and accounts for the presence of particles by imposing a virtual force at the fluid‐particle interface. It creates a very accurate representation of the two‐phase system and, unlike LBM, does not require any calibration (provided that the resolution be high enough). However, IBM is the most computationally demanding methodology and has only been applied to moderately large systems of particles^146^, ^147^ or single particle systems.^148^

Most importantly, for large‐scale, industrial simulations, drag force laws for particle collectives can be derived via DNS.

##### Euler–Lagrange Simulation

When using the Euler–Lagrange Simulation (ELS) for a dense suspension, the details of the flow around individual particles are not resolved (particles are point sources) and the fluid‐particle interaction force has to be modeled. It is a major drawback when suspensions with density ratios close to unity, bubbly flows, or a suspension flow at large particle Reynolds numbers (typically larger than 100) are considered. In these cases, the inertia of the fluid is large compared with the inertia of the particles, but detailed information on the fluid motion is unavailable. Hence, ELS is typically applied to gas‐particle systems. Moreover, models for the drag forces are required, which are only approximations of the real system. Depending on whether DEM or HS is used, either the so‐called “CFD–DEM approach”^99^, ^149^ or the “CFD–HS” approach^150^ can be used. Although the latter has widely been used to study gas‐fluidized particle systems, the former can be applied on a broader scale as it is more flexible with respect to inter‐particulate forces. According to Zhou et al.^99^ and Feng and Yu,^151^ the main advantage of CFD–DEM is that detailed particle‐scale information is obtained, including particle trajectories and forces acting on individual particles.

**Figure 8:**
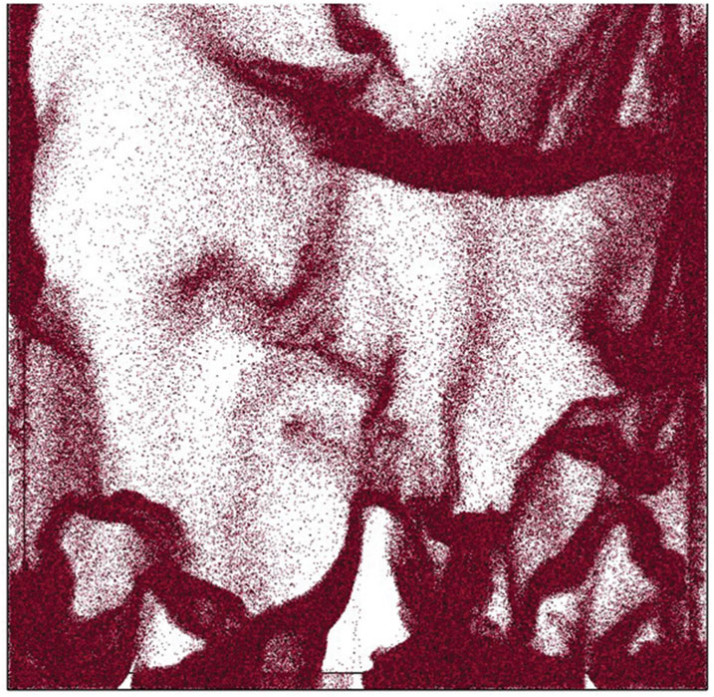
CFD–DEM of a fluid bed process involving 25 million particles. The fine structure of particle clusters (streamers) can be observed. For details regarding the simulation setup refer to Jajcevic et al.^155^

CFD–DEM coupling methods are rarely used to describe pharmaceutical manufacturing processes. Recent pharmaceutical CFD–DEM application include the work of Guo et al.^152^ and Wu and Guo^153^ who studied the effect of air on powder flow during die filling and the analysis of the pneumatic transport of granular media by Sturm et al.^154^ Recently, Jajcevic et al.^155^ reported CFD–DEM simulations of up to 30 million fluidized particles in pharmaceutical manufacturing (see Fig. 8).

A recently proposed method by Sakai and colleagues^156^, ^157^ is based on coupling the DEM and the Moving Particle Semi‐implicit” approach (DEM–MPS), allowing the simulation of suspension flows with free surfaces (DEM–MPS) via fully resolved 3D Lagrangian–Lagrangian simulations.

##### Euler–Euler Simulation

As Euler–Euler Simulation (EES) treats both the fluid and the particle phase as a fluid (some authors refer to EES as two‐fluid models), the details of fluid motion, fluid‐particle interaction, and particle collision dynamics have to be modeled. The advantage of EES is that it does not require tracking of individual particles. Although the theoretical foundation for EES was established by Anderson and Jackson,^158^ research on models for particle collisions is still an active field of fluid mechanics.^159^, ^160^, ^161^, ^162^, ^163^, ^164^, ^165^ However, studies are mostly limited to mono‐disperse spherical particles with only a few exceptions. EES are often used in chemical/pharmaceutical engineering applications as it is the only approach that enables studies of suspension flow in large‐scale equipment. As small‐scale details of the flow are neglected, EES requires rigorous validation and the computational grid size often has a significant effect on the result.^166^ The latter study has triggered the development of advanced EES models that aim at eliminating the grid dependency, some of which were recently created^167^, ^168^, ^169^ (and successfully validated with detailed simulations) for gas‐particle systems, making EES an efficient tool for studying suspension flow in large‐scale equipment.

#### Process Simulation

For many years, simulation of the plant‐wide system behavior, via either static or dynamic simulators, has been a common tool used by process engineers to study and optimize the performance of chemical and petrochemical plants. In the pharmaceutical field, however, it has rarely been used as (1) CM is still only in the adoption phase and (2) process simulators for solid materials have only recently reached the level of sophistication required for routine applications. For example, Parsival, SolidSim, and gSolids are flow‐sheet simulation tools especially designed for solid processing operations, which offer a comprehensive set of additional features (e.g., unit operations libraries, custom modeling, dynamic simulation, physical property libraries) and allow the implementation of kinetic models and coupling with other classical simulation tools (e.g., CFD, DEM, and Matlab). They can model size fractions via so‐called PBE models. Using population balance models to study particle‐population dynamics (i.e., the change in particle size distributions) has become increasingly common in the last few decades. Hulburt and Katz^170^ were the first to present PBEs for a class of problems in particle technology. A large number of attempts have since been made to apply population balance modeling to particulate processes. Several numerical techniques exist for solving PBEs. They were reviewed by Ramkrishna and Mahoney^171^ and Kraft.^172^ In recent years, various studies have been performed with the special focus on understanding the dynamics of the process: time to steady state, the interaction of unit operations, the effect of process upsets, start‐up, and shut sequences and process optimization. Application examples include crystallization,^173^ granulation^174^, ^175^ tablet manufacturing,^176^ and blending.^177^, ^178^ Modeling of continuous process plants were carried out as well, for example, by.^178^, ^179^ Flow‐sheet‐based control models for plant‐wide control via model predictive control (MPC) were recently implemented.^179^, ^180^

In the future, process optimization and control via dynamic flow sheeting will be of increasing importance, especially in the context of CM where these models are crucial for process control and process optimization. However, more robust simulation tools, allowing the standard use of multi‐dimensional PBE and dynamic simulation are required. In addition, modern tools to characterize the dynamics of non‐steady‐state systems, such as bifurcation analysis, should be used.^181^

#### Final Remarks on Process Simulation

Clearly, models are just an approximate representation of reality and are valid only within a certain range of conditions. Materials and process parameters have to be established using sophisticated experimental methods. Accuracy, reliability, and prediction ability have to be established for every model and for every simulation method. This process (termed model validation) is critical, especially for design and scale‐up models. Validation is best performed via (1) simplified setups for which analytical or exact solutions are known, (2) comparison with existing well‐established solutions in the literature, (3) predictions by well‐established simulations tools or experiments carried out at various scales. With that regard, it is important to note that experiments are error‐prone as well. In engineering, an agreement in the range of 10% between experiment and simulation is considered sufficiently accurate for most applications.

Finally, it should be noted that field of modeling and simulation is rapidly advancing. Not only simulation codes are becoming more sophisticated every year and mechanistic models are improved continuously, but also the hardware is developing. For example, a short while ago, a few hundred‐thousand particles were considered the upper limit for DEM simulations. Currently, advanced GPU codes (running on graphic cards) can be used to simulate in the order of 100 million particles. A few years from now even particle numbers above 1 billion may be achieved. Although the area is rapidly developing, ways to combine process models with molecular simulation tools (not reviewed here) and methods to simulate/predict material properties and constitutive relationships in a straightforward manner have to be identified. Thus, significant research efforts are required in the future.

### Materials Science

The chemical compounds used for medication purposes are becoming more complex and, simultaneously, the demand for highly engineered innovative formulations is growing.^182^, ^183^, ^184^, ^185^ As such, the role of materials science is gaining importance.^186^, ^187^, ^188^ Material characterization will be progressively more significant, as explaining the processability of complex systems requires a detailed characterization of the structure of matter. Development of products based on well‐defined solid forms (polymorph, solvate, salt, co‐crystal, and amorphous) of a given low‐molecular‐weight compounds, as well as the complex nature of biopharmaceutical drugs, such as monoclonal antibodies and recombinant proteins, are underpinning the importance of fundamental materials science. Understanding the material properties is a key for successful commercial‐scale manufacturing of pharmaceuticals. Future manufacturing solutions for innovative drug delivery systems (DDSs) can be based on complex and non‐traditional pharmaceutical engineering principles, for example, microfluidics and lithography.^189^, ^190^ At the same time, the success of innovative therapies can be investigated using nano‐level theranostics with increasingly powerful imaging modalities, such as magnetic resonance imaging (MRI), optical imaging, ultrasonography, positron emission tomography, computer tomography, and single photon emission computed tomography.^191^ This review is not aiming to provide a full overview of all available techniques, but we present a few examples of solid‐state analytical tools and related screening approaches.

Techniques that have earlier not been considered as a first choice when analyzing pharmaceutical solid dosage forms are becoming routine: the structure of matter and intermolecular interactions can be explored using solid‐state nuclear magnetic resonance (NMR)^192^ and synchrotron radiation.^193^, ^194^ Via synchrotron radiation, a snapshot of changes in the solid form composition of the sample can be provided in less than a second. Through special sample holder designs, structural changes during dissolution testing have been evaluated. By mimicking the stress conditions occurring at various process steps, a more detailed representation of the solid form composition during processing can be achieved. The implementation of various tools for visualization of the inner 3D structure of dosage forms^195^, ^196^ and novel imaging modalities^197^, ^198^ offers a complete picture of the entire dosage form and not only the surface information. Electron microscope techniques can be coupled with elemental analysis to provide an overview of the spatial distribution of various elements in the sample. Problems related to sample preparation (cutting) can be avoided by utilizing some of the imaging modalities as mentioned above: X‐ray computed micro‐tomography, MRI, imaging at terahertz frequencies and optical coherence tomography (OCT). Innovative thermal analysis^199^ and rheological evaluation of molten polymer–API mixtures can help to design processing conditions for preparation of solid dispersions, for example, with extrusion and 3D printing principles.^200^ Surface‐sensitive techniques probing surface energetics are of critical importance when exploring bulk powder behavior.^201^ There are several methods of quantitative analysis of bulk powder behavior, and powder rheometer is a commonly used approach for troubleshooting in the production environment.^202^

**Figure 9:**
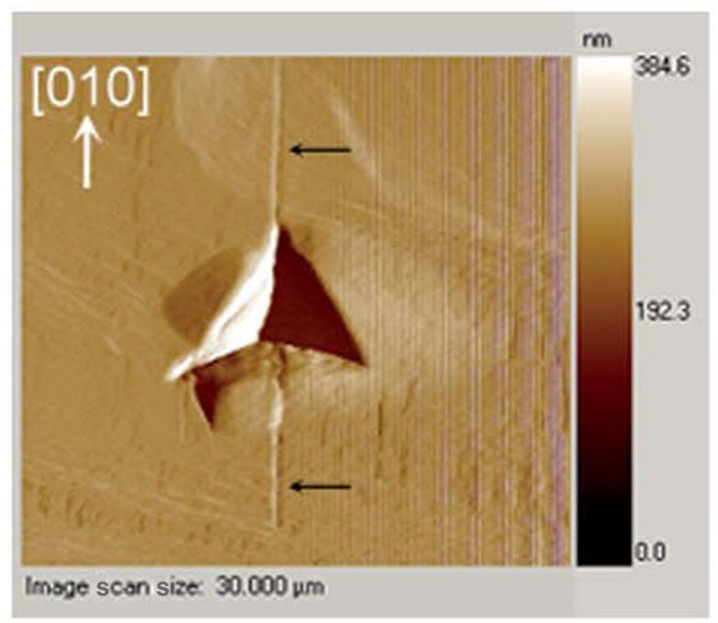
Nanoindentation with atomic force microscope (AFM) helping to identify a weak crystallographic direction in the crystal.^203^ Reproduced with permission of The Royal Society of Chemistry.

Detailed analysis of material properties on a nanoscale can be related to processability of this material (Fig. 9). Single‐crystal level observation with an atomic force microscope (AFM) was directly related to the packing of molecules in the crystal and used to explain the behavior at the particle level.^203^

The number of innovative DDSs on the market is still limited. One of the major obstacles is the lack of scalable manufacturing solutions for DDSs. As drug products administered orally and in the solid dosage form remain the most preferred solutions, it is important to develop these complex formulations with acceptable particulate properties. The size and shape of particles for the final product can be designed from the molecular point of view.^204^, ^205^ Both top‐down and bottom‐up approaches have been suggested for optimizing the bulk characteristics of starting materials.^206^ Bottom‐up particle design approach via, for example, controlled crystallization could be implemented already during the final purification phase after primary manufacturing (synthesis).^207^ Bottom‐up approaches can follow different strategies with a specific aim to control both the crystallization of a desired solid form and the particle morphology with desired surface characteristics. Tailor‐made additives can be effective for achieving the optimal material for downstream processing. Control of intermolecular interactions can be based on a strategic selection of polymers with desired functionality and on advances in understanding of the mechanisms (e.g., of polymer‐induced hetero‐nucleation, indicating the potential of this approach^208^). Computational work can contribute to this approach, for example, in investigating solvent effects on the morphology^209^ and in estimating computationally the additive‐active ingredient interactions.^210^, ^211^, ^212^ Small molecules, such as water, can also be used as a design element in materials engineering.^213^ The role of excipients should also be revisited, and excipients (or additives in the engineering language) can become a more natural part of up‐stream processing in the future.^214^ This way, less processing steps will be involved in producing a material with an acceptable performance for the final dosage form. Materials with appropriate bulk characteristics (flowability) are required when implementing innovative engineering for processing of the final product.

Solid‐form screening is a normal part of drug development activities and several strategies and platforms on different scales do exist for this activity.^215^, ^216^, ^217^, ^218^, ^219^ Significant progress has been made in the computational prediction of solid forms during the past decade. A good indicator of the development in this field is the crystal structure prediction blind test series organized by the Cambridge Crystallographic Data Centre.^220^ The recent fifth blind test showed that although crystal structures of relatively rigid and slightly flexible small molecules can be predicted, more work is required for larger, more flexible molecules and complex systems, such as salts and hydrates. Because of the continuously improving performance of computer systems and force fields, several computational groups can now predict the crystal structure. The challenge is to relate these computational findings to practical pharmaceutical formulation development or for the prediction of complex phenomena, such as stability or dissolution.

**Figure 10:**
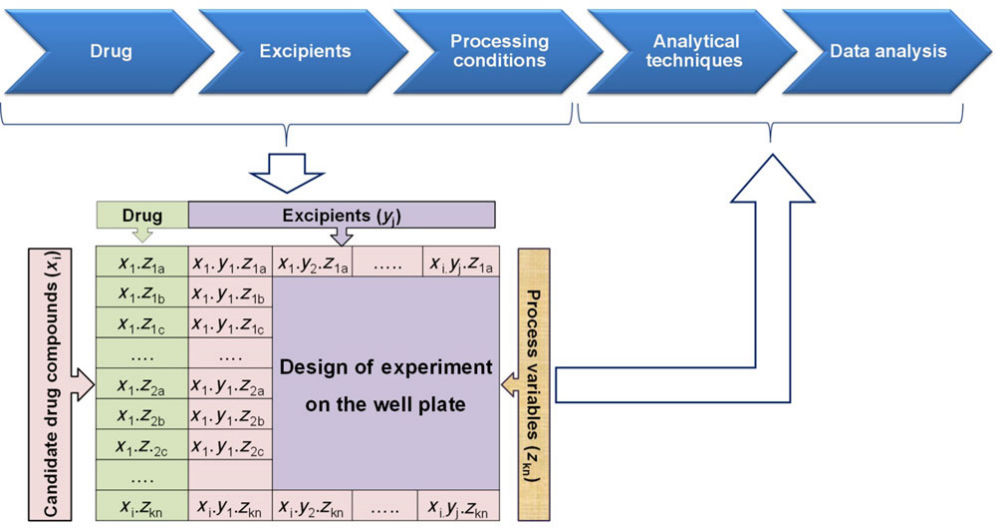
Small‐scale formulation screening platform for estimating the role of secondary manufacturing operations on solid form stability.^223^ Reprinted with permission from Elsevier.

Handling high numbers of samples has been made possible by technical innovations in the field of robotics and high‐throughput screening (HTS) and related analytics, for example, Raman spectroscopy. One difficult aspect of experimental solid form screening is the small‐scale handling of solid matter (weighing and transport of powder in various HTS geometries). Efficient solid‐form screening also requires fast solutions for estimating the practical importance of the identified new solid form,^221^ support tools for decision‐making concerning the optimal final dosage form and the related manufacturing solutions^222^, ^223^ and, further, innovative analytical tools for clustering the experimental results.^224^ Figure 10 illustrates an experimental platform for evaluating the role of excipients in the development of solid dosage forms and decision support for final process solution.

An important part of the bottom‐up‐based materials engineering approach is monitoring and controlling the solid form composition of the product during manufacturing in order to achieve the desired fine‐tuned clinical response. When performing the process design for potential future dosage forms consisting of carefully designed high‐tech materials, it becomes increasingly important to utilize appropriate process analytical tools during manufacturing.

## PROCESS ANALYSIS FROM THE ENGINEERING POINT OF VIEW

### Process Measurements

#### Process Interfacing

**Figure 11:**
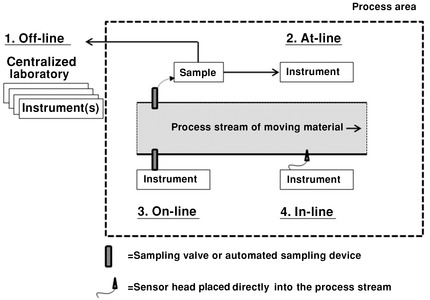
Schematic illustration of various modes of process analysis. Modified from Ref. 5.

An important starting point of implementing process analytical solutions is interfacing with the materials under investigation (Fig. 11). Collecting the representative signal can be ensured by a proper consideration of the placement of analytical instruments and, especially, the placement of a sensor/probe head. Interfacing can be performed in several ways:

- at‐line,
- on‐line, and
- in‐line.

The term *off‐line* is used to describe a situation in which samples are removed from the process stream and taken to a centralized lab located outside the processing area. This is the optimal solution from the analytical point of view, which implies a centralized location of the instruments and expertise. However, if the obtained information is to be used for process analysis decision‐making or real‐time quality control (QC), time gap between sampling and receiving the results might be too long.

The other extreme, *in‐line* analysis, is used to describe a situation in which the probe head is directly inserted into the process stream. Because of the challenges related to cGMP, it is not always a preferred solution and can especially be difficult with regard to biotechnological processes (sterility issues).

Between these two extremes is *at‐line* or *on‐line* analyses, which involve removing a sample from the process stream but analyzing it in the process area. *At‐line* analysis is performed by manually taking the sample to the measuring instrument and, in many cases, not returning it to the process stream. *On‐line* analysis often involves automated sampling and returning it to the process stream.

Practical issues, such as fouling of the sensor/probe head, can become a major hurdle for the implementation of process analytical solution. Different sampling solutions for *at‐line/on‐line* analysis and process window solutions for *in‐line* measurements can be a part of commercial process analytical solution. Many of these solutions involve purging gas or mechanical removal of the material disturbing the process measurement. Innovations in this area have been reported in the literature.^225^

Process measurements from liquid phase process streams are well‐established. Interfacing with liquid phase systems and sampling, for example, from synthetic reaction streams for HPLC analysis, create other challenges than interfacing with solid‐state samples. When interfacing a process probe with a moving powder/tablet stream, several additional issues related to powder handling can arise. Because of the segregation tendency, a wrong placement of the probe can lead to a completely misleading analytical result. Interfacing decisions must also be based on the flow dynamics of the solid material under investigation and the process dynamics.^226^, ^227^ Sampling frequency of the measurement should be realistic in terms of the measured phenomena, for example, monitoring of a drying process does not require a millisecond scale sampling frequency, but fast chemical reactions may need fast analytics. Finding an optimal place for the probe head can be facilitated by computational approach and process simulations.^228^ Another strategy would be to increase the number of measurement points and multiplex several probes (e.g., perform multipoint NIR measurement).^229^, ^230^, ^231^

Several process measurements involve complex physical interactions of light and material (e.g., scattering in spectroscopic measurements, diffraction for particle size determination). These interactions have been evaluated theoretically.^232^ In practice, the required sampling volume needs to be estimated for successful measurements.^233^, ^234^ A good example is Raman spectroscopy, with several practical probe‐design factors are affecting the collected signal. Probe‐head optics can be used to optimize the effective sampling volume and make sure that the collected signal represents the whole dosage form.^235^ Raman spectroscopy is associated with a challenge related to a possible energy input from the measurement itself—intensive radiation from the laser can induce degradation of components of the formulation. These examples highlight the importance of carefully considering the physical principles of the measurement technique and optimizing the measurement solution for the intended use.

#### Sensor Technologies and Related Data Management

Careful selection of a right sensor for a specific process analytical task is key for successful process monitoring and control solution. Robust processes can be developed based on relatively simple (univariate) measurements, without necessarily requiring a high‐end expensive process measurement solution, for example, spectrometer. Standard measurements, including temperature, absolute/relative humidity, pressure, mass, force, and torque, are elements of well‐established engineering methods that should form a basis for all process analytical work. The investment into expensive and more complex process analytical tools should be based on risk assessment and a well‐documented need. There is a wide variety of complex process analytical tools opening a possibility for totally new manufacturing solutions and regulatory philosophy. Interfacing a continuously operating manufacturing line with the correct process analytical tools will constitute a fundamental change in the pharmaceutical field.

Current developments in process analytical chemistry have provided new insights into manufacturing of pharmaceuticals. Several spectroscopic techniques are widely used^236^, ^237^, ^238^: during the last decade, process spectroscopy using IR, NIR, and Raman have been well described for several applications and have becoming widely accepted in the industrial setting. Moreover, novel methods and combinations have been proposed, for example, the combination of Raman spectroscopy and dynamic light scattering for the characterization of therapeutic proteins.^238^ Spectroscopic methods have been described in pharmacopoeia and increasingly accepted by regulatory side. As such, implementation of these methods in real life application should be relatively straightforward.

One of the simplest methods, that is, normal visible light image information, is not as widely utilized as in many other industries. Optical imaging with various innovative configurations has been applied for capturing information related to powder behavior.^225^, ^239^, ^240^, ^241^ Imaging performed with the help of other wavelength regions and chemical imaging using the above‐mentioned techniques (IR, NIR, and Raman) are becoming increasingly popular.^242^, ^243^, ^244^ Chemical imaging has potential in the production environment, and NIR imaging of continuous wet granulation line confirm that this approach is suitable for residence time analysis and mapping of moisture within the moving material.^245^ Fast imaging of moving freeze‐dried biological samples with the similar NIR setting enabled the visualization of moisture distribution and the detection of moisture‐ induced crystallization of the excipient in the formulation.^246^ Imaging of chemical components and solid form variation in moving dosage units can offer new opportunities in QC in the pharmaceutical environment. All this image information can be used as a part of machine vision system to create innovative process control solutions.

A wide variety of other process analytical tools exists, and NIR spectroscopy should not be viewed as the one and only PAT solution. High‐end analytical chemistry tools, including electrochemistry,^247^ chromatography, mass spectrometry, and NMR,^247^ can be considered if risk assessment indicates so. From a more physics‐based‐methods standpoint, diffraction methods have commonly been utilized for particle size determination,^248^ and several other techniques can capture particle size related information as well.^249^ Information from the process can also be extracted using innovative approaches, such as acoustics,^250^, ^251^ ultrasound,^252^ and electrostatic monitoring.^253^ Another example is OCT for monitoring inline the coating thickness and the inter‐ and intra‐tablet coating variability during the coating process (both in pan and fluid‐bed coaters).^254^ These examples were given to provide an idea of the existing possibilities and demonstrate that everything can be measured. With regard to implementing new process analytical sensors, the possibilities are unlimited.^255^ Although it may be tempting to implement several analytical tools in a given unit operation, in reality, the optimal solution can be a very simple system with only a few univariate sensors. However, this may not always be true, and increasing amount of data has to be properly analyzed.

Advances on the sensor side together with the increased interest in hyphenated (combined) techniques and chemical imaging necessitate robust data analytical tools that are capable of handling complex information.^256^, ^257^, ^258^, ^259^ Multivariate data analysis (MVDA) and chemometrics are the terms used for describing activities related to establishing the relationship between the complex analytical signal (e.g., spectrum/spectra or image) and the investigated quality attribute. MVDA is becoming a generally accepted and widely applied technique in the pharmaceutical sciences for both qualitative work (e.g., classification of raw material using principal component analysis, PCA, NIR/Raman spectra from handheld instruments) and quantitative examples (e.g., measurement of water with NIR). The real challenge in the implementation of MVDA is when data are structured in a multi‐way manner,^260^, ^261^ for example, fluorescence emission spectra measured at several excitation wavelengths. Processing of pharmaceuticals involves complex multicomponent systems and, in many cases, requires hyphenated techniques to provide an insight into the system and the implementation of multi‐way data analysis. Although this area of science is developing fast, more work is necessary to secure the development of appropriate standards in the pharmaceutical sector, ensuring implementation of MVDA as a part of future quality system. Implementation of a multivariate method requires critical evaluation of the model, and possible pitfalls have recently been described.^262^, ^263^

Miniaturization of analytical instruments has great potential in the pharmaceutical field. Hand‐held spectrometers can be carried in a pocket of a process operator, adding a new dimension to QA. While integrating hand‐held analytical devices and electronic notebooks into quality systems will be one of the future challenges, it appears to be an attractive option for flexible manufacturing solutions. Implementation of movable analytical devices providing information on moving samples to existing QA/QC and laboratory information management systems requires some fundamental rethinking. Another challenge is the maintenance of a multivariate method, which may require a model/data transformation in order to make it/them compatible with multiple analytical instruments. Several data transformation, model update and systems robustness evaluation methods have been described in the literature.^264^

These developments necessitate improved overall data management solutions and database structures.^63^ One of the major motivating factors and a key driver for using PATs is real‐time release philosophy. Increasing the load of information with a time label can be created to describe one specific batch or, in the case of a continuously operating manufacturing line, be utilized to fulfill the regulatory compliance requirements.

### Process Control Strategies

**Figure 12:**
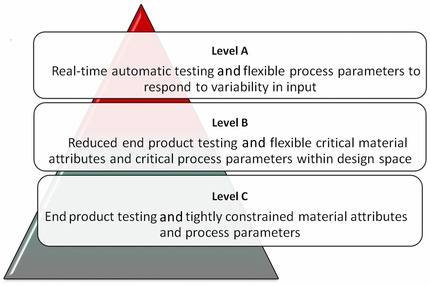
Various levels of control strategies. Adapted from Ref. 269.

Shifting market demands and the trend toward CM require novel control strategies for pharmaceutical production processes.^179^, ^265^, ^266^, ^267^ While control configurations and controller tuning for single batch units are rather straightforward, integrating several unit operations into one continuous plant creates highly complex interactions and dependencies.^268^ Possible solutions include operator‐based control strategies or automated control systems that act directly on the process. In general, a process control system has to have three major features: (1) fast and controlled startup and shutdown, (2) continuous satisfaction of all CQAs by achieving the steady state, and (3) assurance of quality regardless of disturbances, dynamics, uncertainties, nonlinearities, and constraints. The three quality levels of control strategies (see summary in Fig. 12) increasingly depend on real‐ (or almost real‐) time data signals and mechanistic process understanding.

In this context, the choice of the right monitoring tool is important and often challenging, for example, in the case of complex multivariate models for spectroscopic data acquisition. Well‐placed PAT sensors and probes are key elements of an efficient control strategy, enabling CM and possibly real‐time release. In addition, manufacturing‐related data have to be provided for many reasons: to enable QA and QC functions, to satisfy regulatory requirements and to provide the basis for trouble shooting or future formulation development as part of a knowledge management system.

**Figure 13:**
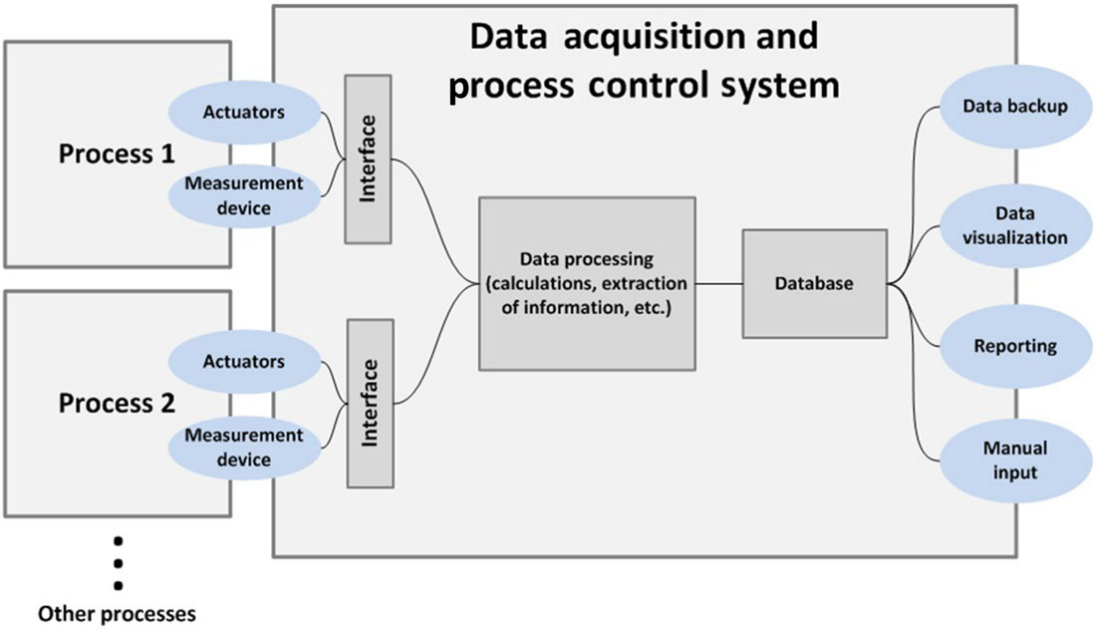
Schematic structure of an automation system (adapted from Ref. 270). The data obtained from the measurement devices are collected and processed in a data acquisition and process control system. The resulting controller action is implemented in the process through suitable actuators. Process data are stored in a comprehensive database for quality assurance and regulatory purposes.

In general, the modular structure of a typical automation system has multiple levels. First, selected measurement devices (probes) have to be physically connected to the process stream at the desired level (*at‐*/*on‐*/*in‐line*) (Fig. 13). Next, an interface enables the transmission of the obtained data to a data acquisition and process control system that collects and processes the measurement information. Finally, the control system returns the controller action to the process, using actuators that adjust the process accordingly.

In order to conform to quality requirements and regulatory demands, both data collection and structure of the associated knowledge management system are critical for an automated control system. Real‐time release and storage of all relevant process information are especially important. With that regard, newly arising issues, such as traceability of the pharmaceutical product throughout the continuous process stream and storing processed data rather than raw data (e.g., the spectrum‐derived API content over time vs. the time‐resolved spectral data), still have to be resolved to avoid conflicts with the regulatory requirements.

**Figure 14:**
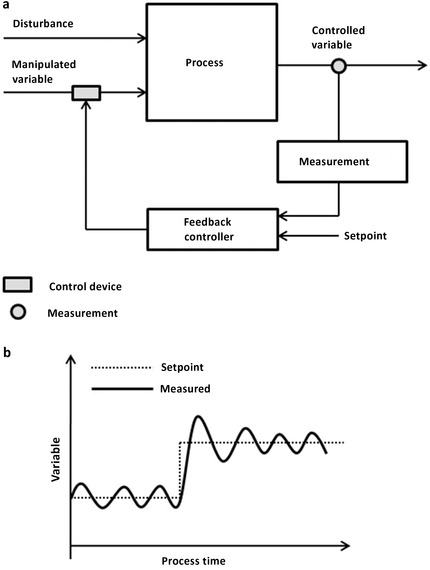
(a) Schematic structure of a feedback control loop. The deviation between the controlled variable and the specified set point is used to trigger and actuator action, adjusting the associated manipulated variable and compensating for the occurring disturbances. (b) Exemplary control action resulting in an overshoot reaction and oscillatory behavior during the set point changes.

The centerpiece of the control system is the controller structure that initiates corrective actions based on the provided measurement information. Control theory suggests various control configurations, for example, feedback, feed‐forward, and cascade control to name a few. A typical feedback control structure is shown in Figure 14a. It constantly calculates the difference between the variable that has to be controlled and a specified set point value (i.e., the error). This error is then processed and forwarded to an actuator that manipulates a correlated process variable (manipulated variable) accordingly. As demonstrated in Figure 14b, desired set points may not be reached immediately or exactly. However, rise and settling time, as well as oscillations around the desired set point, can significantly be reduced and adjusted according to specific process requirements by accurate tuning of the controller parameter and appropriate expertise in the controller design.

In contrast, feed‐forward controllers constitute an open loop control system by triggering an actuator action based on a predefined set point and without considering or monitoring the current status of the controlled variable. Controllers of this type require excellent knowledge of the controlled system and all occurring disturbances so that adequate actuator actions are performed to keep the controlled variable within its acceptable limits. Cascade controllers combine two or more controllers in master and slave loops to serve control variables that interact on various time scales, e.g., faster local stabilization loops and slower supervisory control loops. Proportional (P), differential (D), and integral (I) control terms and combinations thereof are the simplest and most commonly used controller types for determining the control action of the manipulated variable. PID control is considered to be the best option for a general process with unknown dynamics and optimal for serving fast control loops.

Unlike PID control, fuzzy controllers evaluate physical input signals with linguistic terms gained from human expert knowledge via logic of operations.^271^ This allows to process fuzzy process knowledge and expertise and to transform them into precise actuator settings for the automated control purposes.

**Figure 15:**
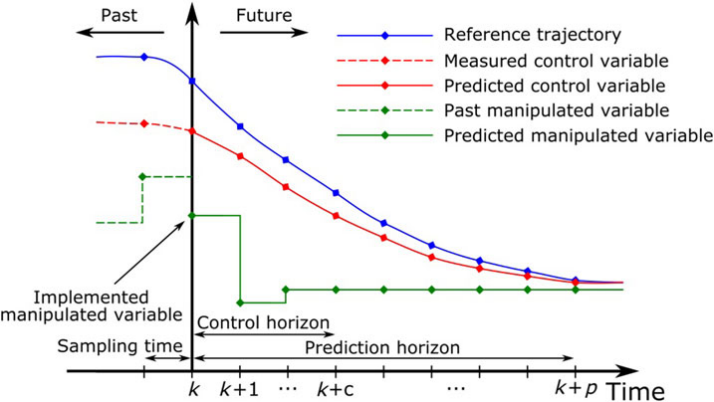
Schematic structure of a MPC approach (adapted from Ref. 265). Past measurements of control variables and prior implemented actuator values are used to predict the future behavior of the system.

In industries, in which automated process control is an inherent part of process development,^176^ advanced controller approaches, such as MPC, have been applied for many years. The pharmaceutical industry is beginning to introduce such methods as well.^265^, ^272^, ^273^ MPC is especially suitable for multivariate problems with difficult dynamics and large time delays, in conjunction with certain input/output constraints. It calculates future actuator values using a dynamic presses model (either mechanistic or stochastic) and past and current measurements. A schematic of the control scheme is shown in Figure 15. The future actuator values, spanning the so‐called control horizon, are determined such that the predicted values of the controlled variables are approaching the desired target values over the prediction horizon. It is achieved by solving a minimization problem of a defined objective function fulfilling all given process constraints. A distinguishing feature of MPC is that even though the control horizon comprises several actuator steps, only the first control action is effectively implemented. For the next action, future values of the new control horizon are recalculated and, again, only the first control action is implemented. This is the reason why MPC is also referred to as the “receding horizon approach.”^274^ With regard to pharmaceutical CM, the computationally extensive MPC can be integrated into a supervisory control layer as time delays between control action and the effect on the product quality may be greater, for example, in the order of 10 min to 1 h.

Most current publications propose simple and common proportional–integral(–derivative) [PI(D)] control systems for pharmaceutical production plants.^179^, ^266^, ^267^, ^275^, ^276^, ^277^, ^278^ Only a few studies have so far reported a successful development of an MPC or MPC‐hybrid control structure for simulated processes.^265^, ^272^, ^273^, ^278^, ^279^ Since several works indicated unsatisfactory performance of control approaches based on PID, advanced process control structures, such as MPC,^267^, ^275^, ^277^, ^278^, ^280^ are generally recommended.

An interesting method was proposed by Rolandi and Romagnoli in 2005 and 2010.^281^, ^282^ Even though it was designed for the chemical industry, their MPC for on‐line full optimizing control may encourage future developments in the pharmaceutical industry. A similar process‐adaptive approach was reported by Singh et al. in 2013^265^ who updated model order and corresponding coefficients of a linear MPC online via system identification after a certain run time interval to comply with current process dynamics and make more precise predictions. A combination of scheduling and control via MPC was reported recently.^283^

In the end, the choice of a control structure depends on the specifics of the process and a consideration of all control expectations and requirements. In some cases, a combined approach may be most suitable. Such combined structures can vary from simple cascaded PI(D) controllers, serving faster and slower control loops and interacting process variables at a time,^266^, ^267^ to novel MPC–PID hybrid approaches where MPC represents a supervisory control layer that delivers the set points for faster acting regulatory PID loops.^265^ This is especially beneficial, as the MPC algorithm requires a certain computational time that increases with the complexity of the model and, therefore, cannot serve faster control loops. Furthermore, it depends on accurate, multivariable, and linearized models of the process that might not always be available.

In order to facilitate the implementation of automated control systems in the pharmaceutical industry, in 2008 the International Society for Pharmaceutical Engineering (ISPE) released the Good Automated Manufacturing Practice guideline (GAMP® 5). It provides a pragmatic industry guidance to understanding and risk management of computerized systems in GxP environments, ensuring the identification, analysis, evaluation, and control of associated risks.^284^

Regulatory authorities play an important role in promoting automated CM. Although, in the past, issues such as traceability, real‐time release, recalls, and documentation requirements were addressed, today such principles as the “proposed operation conditions” have to be considered and a tight control of intermediate quality attributes has to be established rather than keeping process parameters within a certain design space. Moreover, appropriate training of technical staff should to be encouraged, and a larger number of joint projects of industry and academia are required to reduce regulatory risks and attain regulatory clarity for industry.^268^

In summary, the industry is moving (slowly but surely) toward automated plant‐wide control systems in standard production. This task is not impossible. Reconsidering obsolete process development approaches and regulatory demands are critical for its success. Advanced automated process control is a critical issue for automated CM and more research in this field is required.

## PERSPECTIVE TO FUTURE PROCESS PHILOSOPHY

### Future Manufacturing Technologies

Over the last years, decade‐old paradigms of pharmaceutical and bio‐pharmaceutical manufacturing have changed dramatically, as regulators, industry, and pharmaceutical scientists began to realize that new product generations could not be produced using outdated technology. Future products are more complex. They are structured on many levels including nano‐structures, typically involve (combinations of) highly active substances at low concentrations and are administered in novel ways. At the same time, higher and higher quality demands and an ever‐increasing cost awareness require effective and robust solutions. Thus, new production technologies will augment classical routes more and more. The main drivers of new technology include (1) CM, including QA in real‐time via PAT, (2) processes suitable for nano‐structured DDSs, and (3) manufacturing technology for individualized and on‐demand drug products. Especially the last issue should not be underestimated. Personalized and individualized medicines, including drug products for specific patient populations (e.g., the pediatric and geriatric patients) and combination products, will rapidly change the pharma landscape. Process engineers will have to provide solutions for future individualized demands. In the following sections, we provide an overview of current and future trends.

In contrast to batch manufacturing, CM establishes a continuous flow of material exposed to a sequence of time‐invariant unit operations, which is constantly monitored and controlled by in‐line analysis tools to ensure that the final product complies with pre‐defined quality attributes. Several advantages are associated with CM, and flexibility is a major one: new processes can be developed faster using the existing CM lines. Moreover, it contributes to the industry's response capacity in case of emergencies by reducing the manufacturing time and the increasing or decreasing the amount of material produced, depending on current needs. Another important advantage is speeding up the supply chain. Existing supply chains may require a few months or even a year or longer, reducing the ability to react to changing market demands (such as epidemics). Long supply chains also complicate the clinical development stage. In addition, CM can reduce scale‐up problems as development can be performed using the manufacturing equipment. By eliminating scale‐up, which may become a significant obstacle on the product's path to market, CM enables a more agile manufacturing process that can quickly be adapted to changes in the demand. During CM CQAs are monitored in real time, improving the product quality. As CM plants have a small footprint, they can be setup in flexible and portable environments, for example, containers, which can be shipped to a specific location (e.g., in developing countries) and have a wide range of applications (e.g., local epidemics, military use, space travel). Intermediate storage and stockpiling can drastically be reduced. In the area of primary manufacturing, more selective catalytic routes and much faster, more exothermic and more elegant chemistries can be applied, involving unstable intermediates or products, high pressures or temperature extremes (e.g., organo‐metallic reactions, nitrations, halogenations, and diazo reactions). Having a low environmental impact and being a source of high‐tech jobs in various regions, CM has a positive effect on the society. Moreover, it helps to reduce the cost of drugs and their development, benefiting the healthcare system and potentially enabling more investment in new products. Using CM, a much wider range of novel dosage forms can be developed and a wider range of doses can be manufactured without extensive alterations to the process.

**Figure 16:**
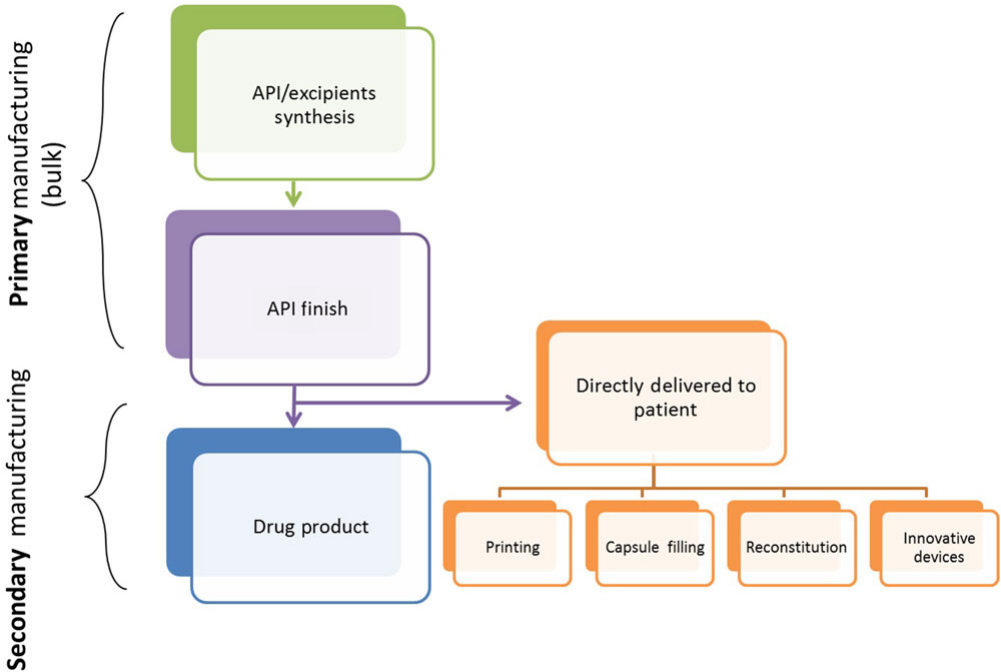
General overview of a continuous manufacturing process, from primary manufacturing (API synthesis, purification, and finish) to secondary manufacturing. It is also possible to make individualized products directly for the patient in a continuous manner.

Figure 16 provides an overview of CM in the pharmaceutical field. Different tools are required for API synthesis and API finish (primary manufacturing) and for drug product manufacturing (secondary manufacturing). During the API synthesis, continuous chemical reactors, which are well‐established in other fields, can be used. However, in the multi‐step synthesis of APIs several problems need to be solved.^285^, ^286^ Novel chemistries, which are not “translated” from batch synthesis, are required. Continuous crystallization (API finish) is another critical step in the purification and final production of API crystals. Several research groups studied various systems of continuous crystallizers, such as mixed‐suspension mixed‐product removal (MSMPR),^287^ plug‐flow,^288^ and continuous oscillatory baffled crystallizers.^289^

Modeling of such systems has been reported in the literature.^290^ Continuous filtration and washing (API finish), although well established in other process industries at a large scale have received less attention on a small scale suitable for API manufacturing and in the GMP environment. Unlike small‐molecule APIs, continuous biopharmaceutical manufacturing, including the corresponding purification technology, is still in its infancy. However, several companies such as Genzyme, are taking steps towards CM of biologic drugs. In upstream bioprocessing, perfusion methods advanced from concentrations of 10–15 million cells/mL to of 50–80 million cells/mL. Moreover, several down‐stream options are now available, such as continuous centrifuges, filtration systems, continuous precipitators, extraction, and chromatography, including simulated moving‐bed chromatography that is in an adoption phase for downstream separation and purification.^291^

In the field of solid dosage form, over the last years CM of several industrial systems has been developed (e.g., the GEA ConsiGma™ continuous line, the GLATT continuous granulators and dryers and the continuous blenders for example by Gericke or Hosokawa). Other systems are underway (e.g., by Bohle) and will become available in the years to come. Several academic studies in this field have been performed.^292^, ^293^, ^294^

Printing is another interesting technique, which is currently being developed by several groups.^295^, ^296^, ^297^, ^298^, ^299^ It has several advantages, including the high precision and technological maturity of printing systems, the ability to print complex formulations of various APIs and the potential for on‐demand individualized manufacturing. However, issues, such as speed, robustness, and reliability, remain challenging. Currently envisioned carrier systems range from edible paper to oral thin films that dissolve upon delivery and/or printing of multi‐layer systems. However, more complex formulations and delivery pathways can be realized through printing of drugs. However, several issues need to be controlled and monitored, including the spatial distribution of API, the interaction with carriers, the exact dose and the removal of solvent. Moreover, logistics of the systems and its deployment in hospitals, pharmacies or, in the future, even to the patient have to be addressed.

Although 3D printing is another possible route for manufacturing DDSs, because of the limited speed, it can be used mainly in the manufacturing of drug‐eluting implants, scaffolds,^300^ and medical devices^301^ or special DDSs, such as vaginal rings, dental, or otic drug delivery devices, or other complex 3D products, the simplest being bi‐layer tablets.^302^ The ability to structure a product in three dimensions allows a precise control over the drug loading and release characteristics. 3D printing can be accomplished with powders, melts, and liquids, depending on the characteristics of the carrier material and the API.

Hot‐melt extrusion (HME), although established in other fields for decades, is still a rather novel and innovative process in pharmaceutical manufacturing and is quite promising with regard to advanced delivery requirements. For example, it has high potential to enhance the bioavailability of poorly soluble drugs, which is a frequent and growing challenge in formulation development. Beyond that, the possible capabilities of HME include the achievement of controlled release systems^303^ or the incorporation of nanoparticles in a solid matrices.^304^ Moreover, the continuous characteristics of HME can be beneficial (depending on the application), for example, to achieve high productivity and constant product quality in a cost‐efficient way. For more details about goals and applications of HME, readers are referred to Refs. 270, 292, and 305. In addition, co‐extrusion is increasingly viewed as an interesting tool for developing structured drug‐release systems.^306^ Furthermore, the modeling and online control of HME systems have been reported.^307^, ^308^, ^309^, ^310^, ^311^, ^312^

With regard to pharmaceutical HME, a co‐rotating intermeshing twin‐screw design (Fig. 7) is typically preferred because of its self‐cleaning screw profile and excellent mixing capabilities. A more complex multi screw design is rarely used, for example, for specific applications with extreme devolatilization requirements, as it can achieve a higher specific surface than single and twin‐screws. Downstream equipment includes hot‐die cutters, calandering systems, cold‐strand cutters, and so on.^292^

Similarly to HME, injection molding (IM) is well known in the polymer industry. As well‐defined shapes and sizes can be produced using this technique, it is promising with regard to flexible solid dosage forms in pharmaceutical manufacturing. Because of similar process conditions, the potential to produce solid dispersions and to enhance the bioavailability of poorly soluble drugs is comparable to HME. A recent review of pharmaceutical IM was published by Zema et al.^313^ The IM process is similar to HME in some aspects. First, the granular feed is molten. Mixing is typically not performed using IM, but rather prior to IM in a HME unit. Instead of continuous extrusion during HME, during IM the melt is injected semi‐continuously into the shaping mold under high pressure. The operating pressure during IM can reach up to several 1000 bar (which is not suitable for all APIs) and depends on the shape of the cavity and the rheological properties of the melt. The number of pieces per cycle can easily be adapted via the geometry of the mold cavity. Depending on the shape of the product, it is possible to achieve a quantity of 100 pieces and even more per cycle. The cycle time depends on the material properties of the formulation and is typically in the order of seconds. Thus, a production rate up to 100,000 pieces per hour can reasonably be achieved.

Capsule filling is another old yet innovative process for manufacturing individualized low‐dose drug products for oral delivery or inhalation. However, low‐dose capsule filling is not trivial and only a few systems exist that have reached the technical maturity. They are typically not used for routine manufacturing but are rather applied in small‐scale clinical studies and during development phases that typically involve vibrating capillaries.^314^, ^315^ Examples include the Capsugel Xcelodose micro‐dosing system and the micro‐dosing system by MG2. In both cases, vibrations are used to dose small amounts of powder. In the MG2 system, the fill weigh is measured by an electrical capacitance sensor. Standard capsule filling processes are most likely not precise enough for individualized low dosing. Nevertheless, it can be expected that such approaches will increasingly be used for on‐demand manufacturing of drug mixtures for oral and inhalation delivery.

### Future Healthcare System

The healthcare sector is facing several major challenges: the ageing population and the increased cost of medications for the society require fundamental changes in this business area. The fields of genomics and personal diagnostics have undergone a fast development. The Human Genome Project has created a massive database enabling the development of more tailor‐made drug products and decreased the price of sequencing an average human genome to the $1,000 (Illumina, the leading maker of DNA sequencers announced the $1,000 early 2014). However, all this knowledge has not been translated into commercial success yet.^316^, ^317^ At the moment, oncology is the disease area with most late‐stage development projects.^318^ The recently introduced PMI emphasizes the importance of development in this area, and manufacturing methods for future pharmaceuticals should be modernized now to make this development possible.^20^

There is a gap between the investments into genome research and the final drug product. The research in manufacturing of highly engineered pharmaceuticals has not been acknowledged.^319^ There is a clear need for new manufacturing solutions for the 21st century drug products.^320^, ^321^ Closing the gap between state‐of‐the‐art biology and the final drug product requires focusing more on the innovative pharmaceutical product design. The key enabling factor for cost‐effective personalized therapies is the development of new manufacturing principles. More flexible processing solutions based on continuous operations will enable personalized DDSs with tailor‐made dose, drug release characteristics and combination of multiple drug compounds based on individual needs. All these developments should occur in parallel with the development of genomics and, especially, technological innovations in the field of IT, diagnostic tools, and miniaturized analytical devices.^322^ CM of personalized medicines requires a complete change of mindset in the pharmaceutical business area.

Individual features derived from the genome of a patient can be combined with real‐time diagnostic information from miniaturized analytical devices (Fig. 18). At the patient level, this information can be managed via a portable device (e.g., iPhone) and used for planning a long‐term therapy supported by a feedback from a health care professional. This development emphasizes the need for more flexible manufacturing solutions for the production of personalized drug products.^17^ Manufacturing of all these varying products will be a challenge for the pharmaceutical industry, but more flexible engineering solutions (e.g., extrusion and printing) will make manufacturing‐on‐demand based facilities possible. That will put pressure on the existing distribution chain, which can also be restructured in the case of 100% QC system with real‐time release testing (RTRT). A patient can receive personalized medicines based on manufacturing‐on‐demand not only from the local pharmacy, but also directly from the manufacturer or even by printing them at home. This will change the role of pharmacies in the future. The safety of a medication can be ensured through diagnostic information from the patient and follow‐up using a portable device. This required flexibility can be achieved by implementing fully instrumented CM environment with continuous process/product monitoring and control. As such an environment should be protected from potential hackers, cybersecurity of these systems will be one of the big future challenges, as already identified in the medical device industry.^323^

**Figure 17:**
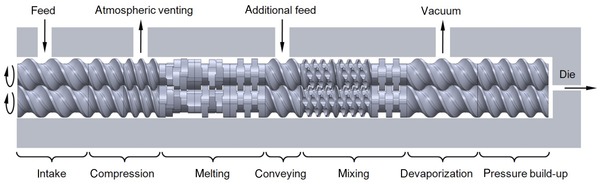
Schematic structure of a co‐rotating, intermeshing twin‐screw extruder.

**Figure 18:**
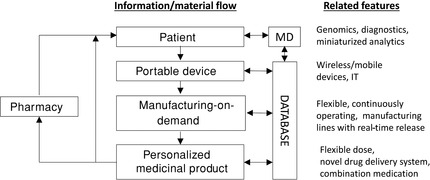
Elements of the future healthcare system.

### Regulatory Framework and Education

Development of engineering solutions in manufacturing sciences puts significant pressure on the existing regulatory framework. Fundamental concepts that were valid during the last century limit the implementation of innovative engineering solutions. Whenever the concept of CM is mentioned, concerns related to the batch concept are immediately raised. Standard pharmacopoeia tests for the variation in the content of an active compound of the final product employ analytical solutions based on wet‐chemistry and off‐line analysis. Today, spectroscopic tools can be used for analyzing thousands of dosage units almost at the production speed. However, in real life, using all this information often causes more regulatory obstacles than improved quality or real financial benefit. It has to be noted that there is regulatory support for the implementation of CM using science‐ and risk‐based approaches.^324^

Recent RTRT guideline and US FDA validation guideline are the examples of positive development in the regulatory field. At the same time, suggestions from the industry^325^ have translated into modified monographs, as in the case of recently adopted Ph.Eur. Monograph 2.9.47, which describes the use of large sample sizes for demonstrating the uniformity of dosage units. All of the above indicates that there is positive attitude toward the paradigm shift in regulatory sciences, which has to be realized as a joined effort of industry, regulatory, and academy.

One of the major obstacles in putting new engineering principles into practice is the structure of educational programs. The global trend in pharmaceutical education programs is to increase the emphasis on biology and social sciences, often at the cost of the “old‐fashioned” pharmaceutical technology discipline. At the same time, several engineering programs have established pharmaceutically oriented programs. Both of these solutions produce candidates with a new type of skill set. This, however, might also have an undesired outcome of having highly skilled engineers from the manufacturing environment trying to communicate with a regulatory person with a more biology‐oriented pharmacist background. Thus, rather than transforming pharmacists into engineers, we should ensure that engineering principles are properly implemented into pharmaceutical education.

## CONCLUSIONS

In this review, we offer an introduction to the toolbox needed for future manufacturing of pharmaceuticals. It demonstrates that in recent years significant progress has been made driven by changes in the regulatory framework (e.g., the PAT initiative and ICH's QbD‐associated guidelines) and a stronger interaction between pharmaceutical and engineering sciences. Moreover, existing gaps with respect to a rational development of drug products and the associated manufacturing processes have become more apparent, ranging from the need to combine molecular, materials, and process models in a comprehensive computational framework to the demand for more advanced PAT tools for certain applications. Although it can be concluded that much of the fundamental knowledge and the technical tools for implementing innovative pharmaceutical manufacturing principles do exist today, more work is required, especially at the interface between pharmaceutical sciences and engineering, essentially defining a new discipline, that is, pharmaceutical engineering science. In summary, the elements required for production of high‐tech future pharmaceuticals have been developed, gaps have been identified and the next step will be a joint effort of academy, industry, and regulatory experts to begin implementing these principles in practice.
